# Assessment tools for medication self-management capacity in community-dwelling older adults with sensory impairment: a scoping review

**DOI:** 10.1186/s12877-025-06768-x

**Published:** 2025-11-25

**Authors:** Eugene Asante, Brenda Morrison, Margaret C. Watson, Marilyn Lennon

**Affiliations:** 1 Department of Computer and Information Sciences, University of Strathclyde, Livingstone Tower, 26 Richmond Street, Glasgow, G1 1XQ Scotland, UK; 2 Strathclyde Institute of Pharmacy & Biomedical Sciences, University of Strathclyde, Glasgow, Scotland, UK

**Keywords:** Medication therapy management, Needs assessment, aged, vision disorders, Hearing disorders

## Abstract

**Background:**

Old age is often associated with an increasing prevalence of sensory impairment (hearing and/or visual) and polypharmacy. This study aimed to (i) identify and describe existing assessment tools suitable for evaluating the ability of older adults to manage medication independently and (ii) report on the validity of the assessment tools identified and applicability to those with sensory impairment.

**Methods:**

A scoping review was conducted and is reported using PRISMA ScR. Electronic databases were searched (PubMed, Web of Science, CINAHL, APA PsycINFO, Cochrane Library) from January 2005 to August 2023. Independent duplicate data extraction was conducted. A manual search of reference lists of all included studies was conducted. There were no restrictions on the country of origin or language. Critical appraisal was undertaken with the Mixed Methods Appraisal Tool.

**Results:**

Seventeen studies were included that reported 17 different tools. Of these, 13 tools assessed some aspects of visual function, often limited to evaluating an individual’s ability to read medicine labels, while only one assessed hearing function. All 17 tools focused primarily on physical and cognitive abilities. Validation was reported with only six tools. None of the tools were specifically developed to assess sensory function/impairment related to medication management.

**Conclusion:**

Given the prevalence of sensory impairment in older adults and its impact on the safe and effective use of medicines, there is an urgent need for validated tools to assess and support the medication-related needs of this population.

**Key Notes:**

• No assessment tools were identified for use with older adults with sensory impairment.

• The majority of the 17 tools included in the review assessed physical and cognitive abilities required for medication management tasks.

• A validated tool is needed to assess and support the additional medication related needs of older adults with sensory impairment.

**Supplementary Information:**

The online version contains supplementary material available at 10.1186/s12877-025-06768-x.

## Introduction

Medication management is an essential personal care activity that many older adults require to maintain healthy, independent living [1]. Capacity for medication management can be defined as the cognitive, functional and sensory ability to self-administer a prescribed medication regimen [2, 3]. Health deterioration, including age-related visual, hearing impairment and dual sensory impairment (hereafter referred to as sensory impairment), often compromises an individual’s ability to manage medications [4, 5]. Sensory impairment can affect an older adult’s independence [6] and well-being [7]. Globally, it is estimated that around one-third of adults aged 65 years and older live with visual impairment, and over 25% of people older than 60 years experience disabling hearing loss [8, 9]. The prevalence of dual sensory impairment also rises steeply with age, affecting up to 10–15% of adults aged 70 years and older [6].

Previous studies have shown that sensory impairment can affect multiple aspects of medicine use. Visual impairment may limit the ability to read small-print medicine labels, distinguish between tablets, or manipulate packaging such as child-resistant caps and eye/ear drop containers, increasing the risk of dosing errors [10, 12]. Hearing impairment can reduce comprehension of verbal medicine instructions provided by prescribers or pharmacists, creating barriers to counselling and limiting the ability to hear reminders or dosing alarms [5, 12]. When both occur together, dual sensory impairment compounds these risks by restricting access to both written and spoken medicine information, further challenging safe and independent medication management [6, 7, 12]. In addition, polypharmacy is more common among older adults with sensory impairment because they have a higher risk of comorbidities compared with older adults without sensory loss, making them vulnerable to medication-related harm [5, 12, 13].

Despite the importance of medication self-management within this population, the formal assessment of an individual’s capacity to manage medication is not performed routinely in clinical or social care practice [14]. Healthcare professionals often rely on patient or caregiver reports, which may lack the necessary depth and detail regarding medication self-management practices [14].

Medicines optimisation was introduced in the United Kingdom to promote a patient-centred approach to the safe and effective use of medicines [15]. It moves beyond a traditional focus on adherence to consider how medicines are prescribed, monitored, and experienced by patients. The Royal Pharmaceutical Society framework sets out four key principles of medicines optimisation [15]:

1. Aim to understand the patient’s experience: recognising that individuals may have concerns, preferences, or barriers to using medicines. For example, an older adult with sensory impairment may find it challenging to identify their tablets, and this concern should be addressed during prescribing and follow-up.
2. Ensure evidence-based choice of medicines: prescribing should be based on the best available evidence and tailored to the individual. For instance, in an older adult with visual impairment, the choice of inhaler device may favour one with clearer dose counters and tactile feedback, rather than a device that relies heavily on visual cues.
3. Ensure medicines use is as safe as possible: regular review of medicines is needed to identify risks such as adverse drug events, drug–drug interactions, or difficulties with administration. For example, the use of child-resistant caps may be unsafe for older adults with reduced dexterity or vision.
4. Make medicines optimisation part of routine practice: embedding medicines review and patient involvement into everyday healthcare. This may include the use of medication management assessments or a multidisciplinary review of complex regimens for older adults with sensory impairment.

These principles emphasise the importance of tailoring medication management to the individual, considering not only clinical evidence but also the lived experience of older adults, including those with sensory impairment.

### Aim

This study aimed to conduct a scoping review of tools used to assess medication self-management capacity in community-dwelling older adults with sensory impairment. For this review, sensory impairment was defined as visual impairment, hearing impairment, or dual sensory impairment, whether partial or complete, and whether acquired or congenital.

### Objectives

The objectives were to:

1. Identify and describe existing assessment tools suitable for evaluating community-dwelling older adults’ ability to manage medication independently.
2. Report on whether the validity or reliability of the identified assessment tools was assessed, and whether the tools could be applied to community-dwelling older adults with sensory impairment.

### Methods

The scoping review was conducted using standardised methods. The protocol was registered with the Open Science Framework, and the results are reported in accordance with the Preferred Reporting Items for Systematic Reviews and Meta-analysis Protocols (PRISMA) [16]. The search period was set from January 2005 to August 2023, following the protocol, which specified an initial start date of 2010 with retrospective extension in 5-year increments if a limited number of studies were identified. Some tools developed before 2005 were still captured through the systematic reviews included in this study, ensuring that earlier assessment tools of relevance were not excluded.

### Eligibility criteria

The Population, Concept, and Context (PCC) [17] framework was used to establish the criteria for including or excluding studies in the review as shown in Table 1. This approach ensured the eligibility criteria were relevant to the review’s objectives and research question.

**Table 1:** Eligibility criteria

Population	Older adults (≥ 65) with sensory impairment. (For hearing impairment, no restriction was placed on how this was defined. Studies were included if they explicitly stated that participants had hearing loss, regardless of whether this was determined by self-report, audiometric testing, or described in the study as a relevant characteristic.)
Concept	Assessment tools designed to assess medicine management in community-dwelling older adults, including those with sensory impairment.
Context	Older adults living at home. Studies focusing on older adults in nursing homes or hospital inpatients will be excluded.

Inclusion criteria

Studies were included that:

- Explored a generic instrument or tool to assess the medication self-management ability of older adults.
- Evaluated individuals’ ability to use medications.
- Involved community-dwelling older adults aged 60 years or older (developing countries) or 65 years or older (developed countries), according to the World Health Organisation (WHO) definition of an older person [18], with sensory impairment.

Exclusion criteria

Studies were excluded that:

- Were not relevant to the assessment of medication self-management.
- Did not consider community-dwelling older adults or people with sensory impairment.
- Included tools that were disease or formulation specific.
- Only considered older adults in an inpatient context.

### Information sources

Electronic databases (PubMed, Web of Science, CINAHL, APA PsycINFO, and Cochrane Library) were searched from January 2005 to August 2023.

### Search strategy

The search strategy included keywords and subject headings (MeSH Terms). The MEDLINE search strategy (Additional file 1) was developed by the research team and reviewed by a subject librarian, with revisions made as necessary. A manual search of the reference lists of all included studies was conducted. There were no restrictions on the country of origin or language.

### Selection of sources of evidence

The search results were uploaded into the Rayyan systematic review software [19] and duplicates were removed. Titles and abstracts were screened by one researcher (EA), with duplicate, independent screening undertaken on a 10% random sample by two individuals (ML, BM). Studies deemed relevant were included in the full-text review. Duplicate, independent screening of the full-text articles was undertaken (EA, BM). Disagreements were resolved by a third reviewer (MW). Where systematic reviews were identified, the primary researcher (EA) identified individual studies within these reviews that fulfilled the inclusion criteria.

### Data extraction

A data extraction table was created. Data extraction was piloted independently by two reviewers (EA and BM) on 10% of the included studies to ensure consistency. Once agreement was achieved, the remaining studies were extracted by one reviewer (EA), with uncertainties discussed with BM. Data was extracted on the characteristics of the publication (title, name of author, year of publication and country of publication); study (aim, design, and sample); and assessment tool (purpose, administrator of the tool, number of assessment tasks, duration of assessment and medication used for assessment).

### Critical appraisal of included studies

The quality of the included primary studies was assessed using the Mixed Methods Appraisal Tool (MMAT) [20]. The MMAT is designed to appraise a range of study designs, including qualitative, quantitative, and mixed methods research, and was therefore appropriate for assessing the primary cross-sectional studies included in this scoping review. Each study was evaluated against five core criteria and rated as ‘Yes’, ‘No’, or ‘Can’t tell’ (the latter indicating insufficient information reported). For cross-sectional studies, these criteria included: whether the research questions were clearly stated, whether the collected data allowed the research questions to be addressed, whether participants were representative of the target population, whether the measurements were appropriate, and whether outcome data were complete.

The systematic reviews identified in this study were appraised using the Joanna Briggs Institute (JBI) Critical Appraisal Checklist for Systematic Reviews and Research Syntheses [21]. This tool evaluates domains, including the clarity of the review question, appropriateness of inclusion criteria, adequacy of the search strategy and resources, the methods used for critical appraisal and data extraction, synthesis methods, assessment of publication bias, and whether recommendations for policy, practice, or future research were supported by the evidence.

The critical appraisal process was piloted using two studies (EA and BM) to ensure consistency, and the remaining studies were appraised by a single reviewer (EA).

## Results

**Fig. 1:**
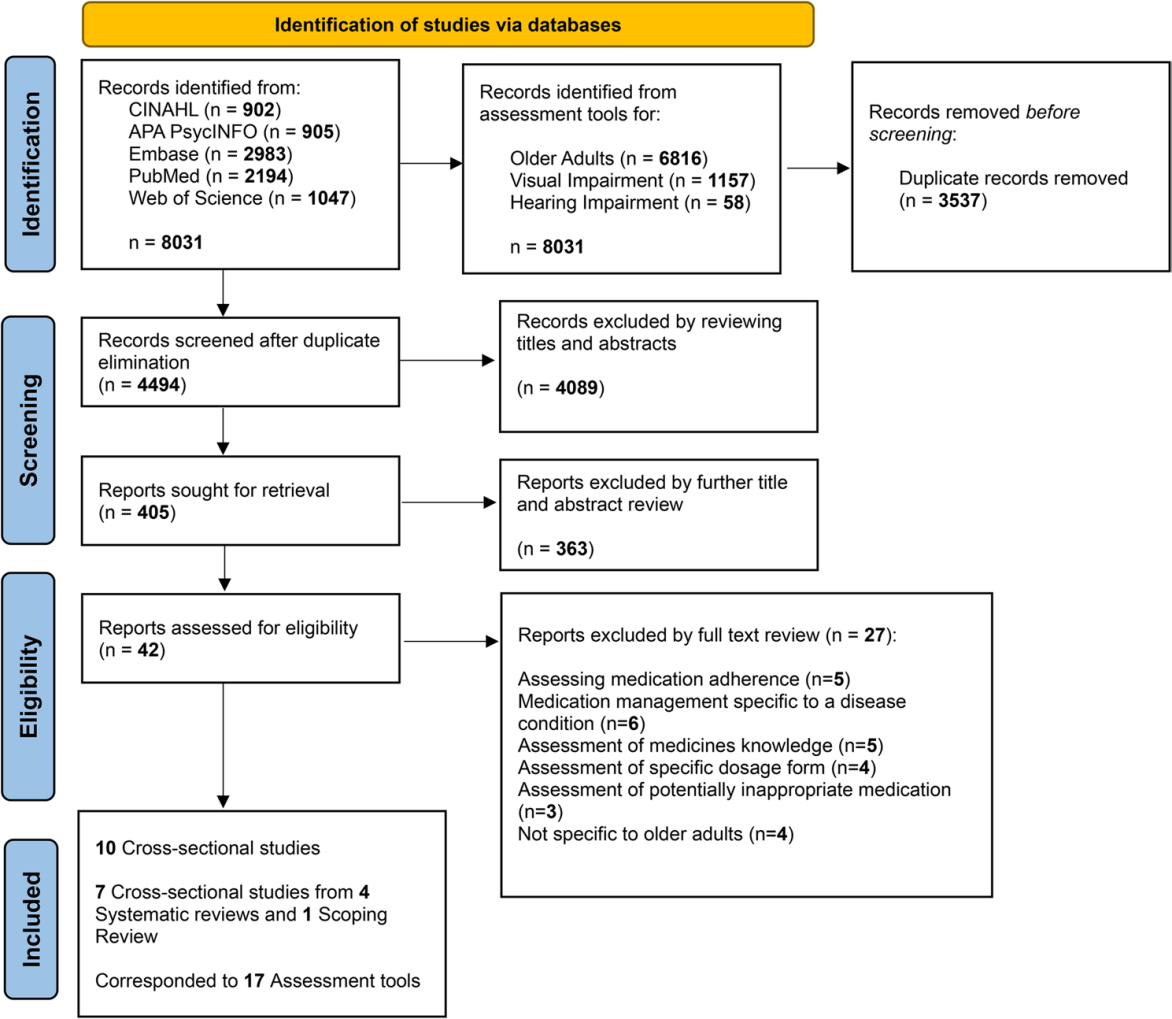
Preferred Reporting Items for Systematic Reviews and Meta-Analysis Extension for Scoping Reviews (PRISMA-ScR) flow diagram

A total of 8031 records were identified across five databases. After removing 3537 duplicates, 4494 records were screened by title and abstract, resulting in 405 full texts for assessment. Of these, 27 were excluded for reasons such as disease-specific focus, adherence-only measures, or lack of relevance to older adults. Ten cross-sectional studies and five systematic reviews were included, corresponding to 17 assessment tools, as illustrated in Fig. 1.

The critical appraisal revealed that all 10 cross-sectional studies met al.l quality criteria using the MMAT [20], indicating consistently high methodological quality. In contrast, the five systematic reviews appraised with the JBI [21] tool met most quality criteria. They lacked assessment of publication bias and independent critical appraisal by two or more reviewers. The complete critical appraisal tables are provided in Additional file 2.

### Study characteristics

Ten [22–31] primary studies were identified through the initial search strategy, and an additional seven studies [32–38] were included from five literature reviews [14, 39–42] identified during the search. It is unclear why the seven studies were not found in the initial search strategy. However, they were included because they met the inclusion criteria and are relevant to the aim of the study. Most included studies were conducted in the United States (*n* = 12), followed by Canada (*n* = 3), the United Kingdom (*n* = 1), and Sweden (*n* = 1) as shown in Table 2. A total of 17 tools [10, 23, 24, 28, 30, 32–38, 43–47] were identified that were relevant for inclusion in this scoping review. Most studies had sample sizes of 30 or more participants, and the average number of daily medications used was reported in seven articles [23, 24, 28, 34, 36, 43, 47], ranging between 3 and 9 medicines. Most of the identified tools [10, 23, 32, 33, 35–38, 44–47] (*n* = 12) were developed between 1986 and 2006, with only three developed after 2010.

**Table 2:** Characteristics of included cross sectional studies

Author, Year of Publication, Country	Assessment Tool	Aim	Sample size, *n*	Mean age in years (± SD)	Average No. of Medications used daily	Test-retest	Inter-item^a^ correlation	Internal^b^ reliability
Edelberg, HK., et al.,[10] 1999 USA	Drug Regimen Unassisted Grading Scale (DRUGS)	To examine an individual’s capacity to manage their medication regimen and standardise the brown bag review.	59	84.20(5.1)	NR	0.9	0.9	NR
Orwig, D., et al.,[23] 2006 USA	Medication Management Instrument for Deficiencies in the Elderly (MedMaIDE)	To describe MedMaIDE and to provide results of reliability and validity testing.	50	78.18 (7.21)	7	0.93	0.74	0.73
Robnett, RH., et al.,[24] 2007 USA	ManageMed	To complete initial reliability and validity psychometric analyses on the ManageMed Screening using a convenience sample of older adults.	67	76	8	NR	0.96	0.89
Irvine-Meek, J., et al.,[28] 2011 Canada	Self-Medication Assessment Tool (SMAT)	To evaluate the psychometric properties and usability.	121	81.5 (7.3)	9.1	0.79	0.83	0.81
Murphy, MC., et al., [30] 2017 USA	In-Home Medication Management Performance Evaluation (HOME-Rx)	To develop a novel, performance-based medication adherence assessment.	5	75.6 (4.4)	NR	NR	NR	NR
Fitten, LJ., et al., [32] 1995 USA	Medication Regimen Assessment Capacity Test (RACT)	To develop an instrument that will facilitate and focus the assessment of a patient’s capacity to adhere to a medication regimen before its initiation.	55	69.95 (7.46)	NR	NR	NR	NR
Hurd, PD., et al.,[33] 1986 USA	Patient’s Barriers to Compliance USA, 1986	To understand the patient barriers to compliance.	14	75.5	NR	NR	NR	NR
Lubinga, SJ., et al.,[34] 2011 UK	Self-Medication Risk Assessment Test (RAT)	To determine scale reliability and validate the instrument against community pharmacists’ assessment of patient’s ability to manage their medications.	37	Median = 76	NR	NR	NR	0.79
Raehl, CL., et al.,[35] 2002 USA	MedTake	To quantify seniors’ abilities to take oral drugs safely and to standardise the brown bag review.	57	79.49 (7.26)	NR	NR	NR	NR
Romonko, L., et al.,[36] 1992 Canada	Pharmacy Assessment (PA)	To develop the PA to better identify drug and patient-specific concerns.	51	80.9	6.5	NR	NR	NR
Schmidt, S., et al.,[37] 2005 USA	Medication Administration Test (MAT)	To examine the construct and concurrent validity.	62	85.56	NR	NR	NR	NR
Stewart, RB., et al.,[38] 1989 USA	Medication Management Evaluation Instrument (MMEI)	To develop a simple objective screening tool that assesses the patient’s functional ability to take medications.	93	74.3 (10.1)	NR	NR	NR	NR
Anderson, K., et al.,[43] 2008 USA	Medi-Cog	To evaluate the association between the MMSE, Mini-Cog, MTS, and Medi-Cog cognitive screens with patients’ ability to fill their own prescribed medications into a pillbox	62	62.5 (13.5)	NR	NR	NR	NR
Carlson, MC., et al.,[44] 2005 USA	Hopkins Medication Schedule (HMS)	To develop and validate the HMS.	360	77.5 (2.8)	NR	NR	NR	0.38
Beckman, A., et al., [45] 2005 Sweden	Medication Management Performance Tests (MMPT)	To develop an instrument that will facilitate and focus the assessment of a patient’s capacity to adhere to a medication regimen before its initiation.	492	82.9	4.08	NR	NR	NR
Isaac, L., et al., [46] 1993 Canada	Self-Medication Task (SM Task)	To describe the development of a method for assessing the relationship between cognitive function, comprehension, and compliance with medication.	20	71.7 (5.8)	3.2	NR	NR	NR
Lamy, PP., et al.,[47] 1986 USA	Medication Assessment Instrument (MAI)	To examine the extent of noncompliance in community-dwelling older adults.	155	71.59 (9.81)	4.5	NR	NR	NR

a - Pearson and Spearman correlations

b - Cronbach alpha NR - Not Reported

### Assessment tool properties

The main goal of the 17 assessment tools [10, 23, 24, 28, 30, 32–38, 43–47] was to quantitatively assess medication management in community-dwelling older adults. The properties of the assessment tools are summarised in Table 3. Overall, the tools lacked a detailed assessment of sensory function for medication management. Most tools (*n* = 13) [24, 28, 32–34, 36–38, 43–47] assessed the ability to read and interpret prescription labels, but only three [33, 36, 47] used different font sizes (small, medium, large) of the label. Five tools [28, 33, 36, 38, 47] evaluated the ability to differentiate between tablets by colour and size. The Pharmacy Assessment (PA) tool [36] was the only tool that measured the individual’s ability to hear medication instructions clearly to perform medication management tasks, as shown in Tables 3 and 4.

**Table 3:** Properties of medication management assessment tools (*n* = 17) (ordered by impairment category)

Assessment Tool	Purpose	Administrator of Tool	No. of Items	Description of assessment	Scoring Scale	Time (min)
Visual + Hearing + Physical + Cognitive Impairment						
Pharmacy Assessment (PA) [36]	To identify barriers to medication self-administration and assist with hospital discharge-planning decisions.	Physicians, nurses & pharmacists	28	(1) Read labels, (2) open vials, (3) remove dose from vials, (4) differentiate tablets by colour, (5) organise pillbox, (6) describe a regimen, (7) swallow pills and (8) Hear instructions clearly	0–28	20
Visual + Physical + Cognitive Impairment						
ManageMed [24]	To quickly determine if someone can handle a moderately complex medication routine.	NR	33	(1) Read prescription labels, (2) Recall information, (3) open/close vials, (4) perform calculations, and (5) organise pillbox	0–42	15–20
Self-Medication Assessment Tool (SMAT) [28]	To screen for medication management deficits in older adults and facilitate targeted interventions.	Pharmacists	44	(1) Read prescription labels in different fonts, (2) recall information, (3) interpret medication instruction, (4) open vials, (5) remove tablets from packaging, (6) differentiate tablets by colour, and (7) organise pillbox	0–28 0–44 (One scoring scale for each section)	NR
Medication Regimen Assessment Capacity Test (RACT) [32]	To develop an instrument that will facilitate and focus the assessment of a patient’s capacity to adhere to a medication regimen before its initiation.	Pharmacists	3	(1) Read prescription labels, (2) comprehend medication regimen, (3) open/close vials, (4) remove dose from vials, and (5) ability to understand medication regimens	0–89	NR
Patient’s Barriers to Compliance [33]	To assess functional abilities that can make compliance difficult for older people.	NR	5	(1) Recall medications, (2) read small font, (3) differentiate tablets by colour and size, (4) open different sizes of vials and liquid containers, and (4) interpret instructions	NR	< 10
Self-Medication Risk Assessment Test (RAT) [34]	To assess elderly patients’ needs for additional support in managing their medications.	NR	13	(1) Read prescription labels, (2) open different medication packaging, and (3) manipulate 5-mL spoon and eye or ear drop bottles	0–26	5–20
Medication Administration Test (MAT) [37]	To aid in placement decisions regarding level of care based on medication management capacity.	Trained non-healthcare professionals	5	(1) Read prescription labels, (2) comprehend medication regimen, (3) open/close vials, (4) remove dose from vials, and (5) organise pillbox	0–100	5–15
Medication Management Evaluation Instrument (MMEI) [38]	To assess the patient’s functional ability to take medication.	Pharmacists	5	(1) Read prescription labels in different fonts, (2) open/close child-resistant and non-child-resistant caps, (3) remove tablets from vials, (4) interpret instructions, and (5) differentiate tablets by colour	0–5	< 5
Hopkins Medication Schedule (HMS) [44]	To screen for medication management deficits in older adults and facilitate targeted interventions.	NR	2	(1) Read prescription labels, (2) comprehend medication regimen, (3) plan a schedule for medication regimen, (4) open/close vials, (5) remove dose from vials, and (6) organise pillbox	0–11	15–30
Medication Management Performance Tests (MMPT) [45]	To identify visual, physical and cognitive barriers in medication management in older adults.	NR	5	(1) Read prescription labels, (2) open vials, (3) interpret medication instructions, and (4) calculate days’ supply	0–5	NR
Self-Medication Task (SM Task) [46]	To assess patients’ medication planning ability and successfully administer a new medication.	Trained research technician	5	(1) Read prescription labels, (2) interpret instructions on labels, (3) open vials, (4) split tablets when required, and (5) organise weekly pillbox	NR	< 20
Medication Assessment Instrument (MAI) [47]	To evaluate patients’ knowledge and skills to take medications and identify barriers to optimal medication management.	Pharmacists	2	(1) Read prescription and auxiliary labels, (2) open different vials, (3) differentiate tablets by colour, (4) name all medications, (5) state indications, (6) duration should be taken, and (7) state dose and time of administration	NR	15–30
Visual + Cognitive Impairment						
Medi-Cog [43]	To assess patients’ ability to fill their own prescribed medications into a pillbox.	NR	3	(1) Read prescription labels, (2) interpret medication instructions, and (3) organise pillbox	0–10	NR
Physical + Cognitive Impairment						
Drug Regimen Unassisted Grading Scale (DRUGS) [10]	To examine an individual’s capacity to manage their own medication regimen and to standardise the brown bag review.	Non-Healthcare Professionals	4	(1) Identify medications, (2) open vials, (3) remove dose from package and 4) state time schedule	0–100	5–15
Medication Management Instrument for Deficiencies in the Elderly (MedMaIDE) [23]	To identify deficiencies in older adults’ ability to take their medication at home.	Non-Healthcare Professionals	20	(1) Medication knowledge (name all drugs and describe complete regimen including indication, route of administration, dose, and time), (2) medication-taking ability (filling a glass of water, sipping enough water, opening vials, removing dose from package, and demonstrate administration method for oral and non-oral dosage form), and (3) knowledge about ongoing supplies (identify existing refills, name of pharmacy or physician office, and available resources)	0–13	30
In-Home Medication Management Performance Evaluation (HOME-Rx) [30]	To assess an older adult’s ability to manage medication routines in the home and to identify at-risk behaviours by home health occupational therapists.	Occupational Therapists	16	(1) Knowledge of medications, (2) recall information, (3) manipulation of medication vials and/or syringes (if used), and (4) calculating medication doses	0–16	30–45
MedTake [35]	To quantify how seniors’ ability to take oral prescription drugs safely may correlate with age, sex, socioeconomic status, education, cognitive impairment, depression, and drug self-management.	Pharmacists & Social workers	4	(1) Identify medications and recall medication names, (2) open vials and remove doses from packaging, (3) state indication, food/water co-ingestion, and (4) dosing time	0–100	30–45

NR - Not Reported

**Table 4:** Comparative summary of medication management assessment tools (*n* = 17)

Assessment Tool	Assesses vision	Assesses hearing	Assesses physical tasks	Assesses cognitive tasks	Reported completion time	Reliability reported	Validation reported
Drug Regimen Unassisted Grading Scale (DRUGS) [10]	No	No	Yes	Yes	5–15 min	Yes	No
Medication Management Instrument for Deficiencies in the Elderly (MedMaIDE) [23]	Yes	No	Yes	Yes	30 min	Yes	Yes
ManageMed [24]	Yes	No	Yes	Yes	15–20 min	Yes	Yes
Self-Medication Assessment Tool (SMAT) [28]	Yes	No	Yes	Yes	NR	Yes	Yes
In-Home Medication Management Performance Evaluation (HOME-Rx) [30]	No	No	Yes	Yes	30–45 min	No	No
Medication Regimen Assessment Capacity Test (RACT) [32]	Yes	No	Yes	Yes	NR	No	No
Patient’s Barriers to Compliance [33]	Yes	No	Yes	Yes	< 10 min	No	No
Self-Medication Risk Assessment Test (RAT) [34]	Yes	No	Yes	Yes	5–20 min	Yes	Yes
MedTake [35]	Yes	No	Yes	Yes	30–45 min	No	No
Pharmacy Assessment (PA) [36]	Yes	Yes	Yes	Yes	~ 20 min	No	No
Medication Administration Test (MAT) [37]	Yes	No	Yes	Yes	NR	No	Yes
Medication Management Evaluation Instrument (MMEI) [38]	Yes	No	Yes	Yes	< 5 min	No	No
Medi-Cog [43]	Yes	No	No	Yes	NR	No	No
Hopkins Medication Schedule (HMS) [44]	Yes	No	Yes	Yes	NR	Yes	Yes
Medication Management Performance Tests (MMPT) [45]	No	No	Yes	Yes	NR	No	No
Self-Medication Task (SM Task) [46]	Yes	No	Yes	Yes	NR	No	No
Medication Assessment Instrument (MAI) [47]	Yes	No	Yes	Yes	15–30 min	No	No

NR - Not Reported

Four assessment tools [10, 23, 30, 38] utilised the older adults’ actual medication regimen, while 10 [24, 32–37, 44, 45, 47] used a standardised medication regimen to evaluate medication self-management. Two studies [28, 46] incorporated actual and standardised medication regimens into their assessment process. Medi-Cog [43] assessed medication management by asking participants to fill a pillbox according to a given medication regimen.

There was variation in completion time across the different tools, with those assessing older adults’ actual medication regimens taking considerably longer (5 to 45 min) as summarised in Table 3. In contrast, tools using a standardised regimen had quicker completion times, most taking less than 20 min. No completion times were reported for Self-Medication Assessment Tool(SMAT) [28], Medication Regimen Assessment Capacity Test (RACT) [32], Medi-Cog [43] and Medication Management Performance Tests(MMPT) [45]. The tools also differed in the number of items or questions, ranging from two to 44, depending on the assessed medication management abilities. The scoring systems also differed, with most tools employing a simple “Yes/No/Unable” format for each assessed task. After completing the evaluation, the total number of “Yes” responses was compared with the total number of medication management tasks, providing a binary judgment of whether the participant could independently manage their medications. No scoring scale was reported for the Patient’s Barriers to Compliance [33], Self-Medication Task(SM Task) [46], and Medication Assessment Instrument(MAI) [47].

The Medication Administration Test(MAT) [37] was administered by trained non-healthcare personnel. In contrast, the Drug Regimen Unassisted Grading Scale(DRUGS) [10] and Medication Management Instrument for Deficiencies in the Elderly(MedMaIDE) [23] were administered by people without formal healthcare training. The SMAT [28], RACT [32], Medication Management Evaluation Instrument (MMEI) [38], and Medication Assessment Instrument(MAI) [47] were all administered by pharmacists. In contrast, MedTake [35] and PA [36] were administered by pharmacists and other healthcare professionals (physicians, nurses and social workers). Home-Rx [30] was administered by a trained occupational therapist in the older adult’s home. Several medication management assessment tools [24, 33, 34, 43–45] did not report information regarding the individuals who administered them or the training required for administration.

All the assessment tools required the older adult to perform a specific task (physical, cognitive, or sensory) and awarded a score based on their performance. They all assessed cognitive function. Twelve tools [10, 23, 24, 28, 30, 32, 35, 37, 38, 43, 44, 46] included an independent cognitive function assessment such as Mini-Mental State Examination (MMSE), Mini-Cog and Neuropsychological Battery to compare medication management scores to cognitive function. Cognitive function was assessed with tasks such as identifying medications, recalling medications, describing medication regimen (indication, route of administration, dose, and time), demonstrating medication administration, and providing knowledge on medication supply. All but one assessment tool (Medi-Cog) [43] evaluated physical function. The most common physical tasks assessed included reading and interpreting prescription labels, opening vials, removing doses from packaging, opening/closing child-resistant caps and manipulating eye or ear drops as shown in Table 3.

### Psychometric properties

Six [10, 23, 24, 28, 34, 44] of the 17 tools reported psychometric testing, primarily focusing on measures of reliability, with limited reporting of validity as shown in Tables 2 and 4. Internal consistency was assessed using Cronbach’s alpha for five tools: DRUGS [10], MedMaIDE [23], ManageMed [24], SMAT [28], and RAT [34]. Reported values ranged from 0.71 to 0.96, indicating high reliability, except for the Hopkins Medication Schedule, which demonstrated low internal consistency (α = 0.38). Test–retest reliability was assessed in a small number of studies, but inter-rater reliability was rarely examined.

Construct validity was reported in some tools, often by correlating medication management scores with established measures of cognitive function, such as the Mini-Mental State Examination (MMSE) or Mini-Cog. Content validity was described during the development of a few tools, but no external validation was undertaken specifically in populations with sensory impairment.

## Discussion

This review aimed to identify medication management assessment tools for community-dwelling older adults and to determine which tools are relevant for use in older adults with sensory impairment. Although 17 assessment tools were included in this review, none were developed and validated specifically for older adults with sensory impairment. In addition, most of the identified tools are dated. Twelve [10, 23, 32, 33, 35–38, 44–47] of the 17 tools were developed between 1986 and 2006, and only three [28, 30, 34] were created after 2010. This indicates that the majority are more than a decade old, limiting their relevance in the context of modern prescribing practices, increasing polypharmacy, and digital health innovations. Only one tool (Pharmacy Assessment) [36] included medication management assessment tasks relevant to both visual and hearing impairments. The range of specific medication management tasks covered by each tool is summarised in Table 5, which illustrates both the variability across instruments and the under-representation of sensory-related tasks.

**Table 5:** Summary of medication management assessment tools and tasks assessed

Assessment Tools	Open bottles/packaging	Read prescription labels	Remove dose from package	Interpret medication instructions	Organise pillbox	Medication recall	Dose/time schedule	Differentiate tablets by colour	Dosage calculation	Medication Identification	Refill information	Split tablets	Hear instructions clearly	Total Medication Management Tasks Assessed
Drug Regimen Unassisted Grading Scale (DRUGS) [10]	✔		✔				✔			✔				4
Medication Management Instrument for Deficiencies in the Elderly (MedMaIDE) [23]	✔		✔			✔	✔			✔	✔			6
ManageMed [24]	✔	✔			✔	✔			✔					5
Self-Medication Assessment Tool (SMAT) [28]	✔	✔	✔	✔	✔	✔		✔						7
In-Home Medication Management Performance Evaluation (HOME-Rx) [30]	✔		✔			✔			✔	✔	✔			6
Medication Regimen Assessment Capacity Test (RACT) [32]	✔	✔	✔	✔										4
Patient’s Barriers to Compliance [33]	✔	✔		✔		✔		✔						5
Self-Medication Risk Assessment Test (RAT) [34]	✔	✔	✔											3
MedTake [35]	✔		✔			✔	✔			✔				5
Pharmacy Assessment (PA) [36]	✔	✔	✔	✔	✔			✔					✔	7
Medication Administration Test (MAT) [37]	✔	✔	✔	✔	✔									5
Medication Management Evaluation Instrument (MMEI) [38]	✔	✔	✔	✔				✔						5
Medi-Cog [43]		✔		✔	✔		✔							4
Hopkins Medication Schedule (HMS) [44]		✔	✔	✔	✔		✔		✔					6
Medication Management Performance Tests (MMPT) [45]	✔	✔		✔					✔					4
Self-Medication Task (SM Task) [46]	✔	✔		✔	✔							✔		5
Medication Assessment Instrument (MAI) [47]	✔	✔				✔	✔	✔						5
TOTAL	15	13	11	10	7	7	6	5	4	4	2	1	1	

### Assessment of vision and hearing function

This review suggests that vision and hearing were rarely incorporated systematically into existing medication management assessment tools. Although some instruments included a label-reading task, very few accounted for the variability in vision (e.g., font size, contrast, lighting), and hearing function was almost absent. This gap suggests that sensory abilities are often treated as secondary to cognitive or physical abilities, despite being significant to safe and effective medicine use. As a result, current tools may fail to identify the medication-related needs of older adults with sensory impairment and provide necessary adaptations or support.

Assessment tools should include the full range of activities in the medicine journey to effectively assess the medication management abilities of older adults with sensory impairment. The lack of a comprehensive evaluation of vision and hearing specific to medication management tasks represents a gap in these tools. Previous studies [4, 14, 42, 48] have highlighted some of the tools included in this review, e.g., DRUGS [10], MedMaIDE [23], MedTake [35] and HMS [44] as effective for assessing general physical and cognitive medication management abilities, but they did not explore vision or hearing abilities. The focus on physical and cognitive abilities might overestimate an older adult’s ability to manage their medication due to failure to explore the impact of their sensory impairment [48].

The assessment tools identified in this review did not reflect Principle 1 (patient’s experience) of the medicines optimisation model [15]. The identified tools may fail to capture the experience of older adults with sensory impairment in relation to medication management because they prioritise physical and cognitive abilities. Understanding patient experience could promote a more individualised and patient-centred approach to medication management in this population.

In relation to Principle 2, “evidence-based choice of medicines” [15], none of the tools evaluated how sensory impairment influences treatment options, prescribing decisions, or the ability of older adults to adhere to evidence-based regimens. Without accounting for the role of vision and hearing in medicine use, there is a risk that clinical decision-making overlooks essential barriers to effective treatment. Incorporating these perspectives would support safer prescribing and better alignment of medicines with patient needs.

The assessment tools in this review did not reflect Principle 3 “ensure medicines use is as safe as possible” [15]. Older adults with sensory impairment are at an increased risk of experiencing adverse drug events compared to older adults without sensory impairment [5]. Safe and effective medicine use relies on identifying and mitigating the unique barriers caused by sensory loss. For assessment tools to align with Principle 3 and ensure safe and effective use of medications, they must evaluate how vision and hearing function impact medication management abilities, such as reading labels, identifying medicines, and interpreting medication instructions accurately.

In relation to Principle 4, “make medicines optimisation part of routine practice” [15], the tools were not designed for use in everyday clinical or community care workflows, limiting their potential for integration into routine medication reviews or multidisciplinary practice. Embedding sensory-specific assessments into routine care could help healthcare professionals identify risks earlier, tailor support, and ensure that medication management strategies are sustained over time for older adults with sensory impairment.

### Duration and administration of assessment tools

The completion times of the included tools ranged from 20 to over 45 min, which limits their applicability in real-world settings for most healthcare professionals and systems [49]. Among the tools reviewed, the In-Home Medication Management Performance Evaluation (Home-Rx) [30] stands out as the only one specifically designed for administration in older adults’ homes by occupational therapists. In contrast, the other 16 tools were developed and validated for clinical settings. While clinical environments offer the advantage of standardised testing, they fail to assess medication management in real-world contexts.

Assessing medication management using an individual’s own medications can provide a more realistic and less stressful experience for older adults, as it mirrors their routine home-based practices [14, 41]. Older adults might be hesitant to bring their medications to healthcare providers due to concerns that poor performance could be perceived as a loss of independence [14, 41]. Although standardised medication regimens facilitate consistent comparisons in research settings by controlling variables, they require significant training and preparation of standardised medication kits, making them less portable and reflective of an individual’s daily medication management. Moreover, these simulated assessments might not accurately capture the influence of an individual’s actual medication regimen or home environment. Factors such as distractions, poor lighting, accessibility issues, and the availability of appropriate aids or devices are difficult to account for in clinical assessments but can significantly impact the ability to manage medication and the accuracy of the assessment tools.

The choice of location for conducting medication management assessments should align with the specific intent and context of the assessment. Clinical settings with standardised regimens may be ideal for research purposes, but some studies [14] have suggested that home-based assessments using an individual’s own medications may provide a more accurate reflection of their capabilities and challenges than assessments using standardised regimens.

### Medication management assessors

Some tools, such as DRUGS [10], MedMaIDE [23] and MAT [37] were designed to be administered by trained non-healthcare personnel. In contrast, several other tools, including the SMAT [28], RACT [32], MMEI [38], and MAI [47], recommend that pharmacists conduct the assessments. None of the tools in this review was administered by the older adults themselves. The involvement of healthcare professionals could improve the quality and clinical relevance of the assessments [50]. However, this would also increase costs and limit scalability compared with non-clinical assessors. Non-clinicians may have limited knowledge of medications, but they could potentially capture the functional aspects of medication management from a “real-world” perspective. Notably, the scoring for many of these assessment tools relies on subjective evaluation of medication management task performance, where assessors must judge which actions constitute a pass or fail. This introduces a potential for inconsistencies, particularly if relying on less-trained assessors. Standardised instructions and adequate assessor training could address these concerns, but were not adequately described across the assessments [51]. Incorporating older adults as assessors themselves could be an innovative approach, promoting self-management and empowerment. This aligns with concepts of medicines optimisation, which emphasises patient involvement in managing their own medications to improve outcomes, enhance safety and person-centeredness in general [15]. Supporting older adults in evaluating their own medication management could provide valuable self-assessment data while promoting independence and adherence to prescribed regimens.

### Psychometric properties of assessment tools

Six of the 17 tools included content and construct validation. Unvalidated tools cannot be relied upon for consistent and/or accurate results [48]. Construct validity was commonly assessed by correlating medication management test scores with measures of global cognitive function tests like the Mini-Mental State Examination. Test-retest reliability and inter-rater reliability evaluations were generally not performed; these are highly relevant for performance-based tests that involve subjective scoring by raters. Without establishing consistent scoring across repeat administrations (test-retest reliability) and between different raters (inter-rater reliability), the obtained scores may not accurately reflect an individual’s actual functional abilities.

### Potential limitations of assessment tools

Most assessment tools focused primarily on assessing physical and cognitive abilities related to medication use. However, other vital factors like motivation and environment may impact an older adult’s overall medication self-management capacity. Psychological aspects, such as health beliefs, and social determinants, such as health literacy, language barriers, and access to community resources, were not evaluated in any of the 17 identified tools. This review also reported only the initial validation studies, without exploring subsequent implementation, clinical utility, or applied practice testing, as these were outside the scope. Since the literature searches were conducted, an additional tool has been identified: the Domain Specific Limitation in Medication Management Capacity (DSL-MMC) tool [3]. This comprises four domains (physical abilities, cognition, medication regimen complexity, and access/caregiver) and 12 subdomains, including vision and hearing. While content validity was established with input from 12 pharmacists, further validation with older adults has yet to be conducted [3].

### Barriers to developing new assessment tools

Developing new, validated assessment tools tailored for sensory impairment remains challenging [52]. Interdisciplinary collaboration across health and social care professionals, gerontology, sensory sciences, and human factors research is essential but difficult to coordinate in practice. Many existing tools were developed for research or clinical trials, and their use in routine care is constrained by time, training, and resource demands. Tools that take more than 20–30 min to administer are rarely feasible in busy health and social care settings [52]. Measuring the extent and severity of vision and hearing loss is challenging, as these conditions vary widely and are hard to assess consistently without standardised tools [7–9]. Limited investment and policy support further hinder innovation compared with better-resourced areas such as cognitive or functional capacity assessments [4, 53, 54].

### Theoretical frameworks on medication management

The omission of sensory domains is notable when considered in relation to established models. The World Health Organisation’s five dimensions of adherence identify patient-related and condition-related factors as determinants of medicines use, both encompassing sensory impairment [55]. Lawton’s model of Instrumental Activities of Daily Living positions medication management as a complex task requiring the interplay of cognitive, physical, and sensory abilities [56]. The Medicines Optimisation Framework also emphasises patient experience and safety [15]. Together, these perspectives demonstrate that sensory functions are vital to an older adult’s capacity to manage medicines safely and effectively.

#### Digital tools for medication management

A growing range of digital technologies, including smartphone applications, electronic reminders, and smart pillboxes, is being developed to support medication management in older adults [57]. These often integrate features such as dosing reminders, regimen tracking, and medication information. However, the assessment tools identified in this review were developed more than a decade ago and focus on cognitive and physical tasks, overlooking sensory abilities. As digital solutions for medication management expand, there is a risk of deploying interventions without considering the accessibility needs of older adults with sensory impairment. Incorporating sensory domains into assessment frameworks would help identify those requiring adaptations (e.g., screen readers, large-font displays, vibration alerts, captioned instructions), ensuring technologies are inclusive rather than creating new barriers.

### Future research

Future work should prioritise the co-design of assessment tools that integrate sensory factors into medication management tasks. Such tools need to extend beyond physical and cognitive function to capture the effects of sensory loss on tasks such as reading prescription labels, hearing instructions, and organising complex medication regimens. A patient-centred perspective, aligned with medicines optimisation principles, should underpin design, ensuring tools reflect older adults’ lived experiences and preferences. Validation should encompass both psychometric properties (reliability, validity) and usability in real-world settings. Research should also investigate accessible digital health technologies that complement or replace paper-based assessments.

### Implications for healthcare

The findings of this review have implications for clinical practice, policy, and public health. Health and social care professionals require tools that reflect the full range of factors influencing safe medicines use, including sensory function. Without such tools, medication-related risks in older adults with sensory impairment may be underestimated, leading to missed opportunities for support. Training should therefore include awareness of sensory barriers and strategies to mitigate them.

From a policy perspective, medicines optimisation frameworks emphasise patient experience and safety [15], yet validated tools accounting for sensory impairment are lacking, creating a gap between policy intent and practice. National guidance on medication safety and polypharmacy should explicitly recognise sensory loss as a risk factor and promote accessible assessment approaches.

At a societal level, failing to account for sensory barriers risks compromising independence, reducing quality of life, and increasing reliance on carers or institutional care. Conversely, effective assessment and tailored support could enable more older adults with sensory impairment to age in place, supporting broader healthy ageing policy goals. The rapid growth of digital interventions further underscores the need for assessment frameworks to guide design, ensuring innovations promote equity rather than deepen digital exclusion.

### Limitations

Grey literature and unpublished studies were not included, so some relevant medication management tools or implementation data may have been missed. Evaluations were limited to the initial validation studies and may not reflect real-world experience with these tools over longer-term use. The analysis of psychometric properties was derived from data reported by the individual study authors and did not involve independent re-scoring of assessment tools. Our primary search strategy began in 2005, in line with the protocol. While this means that some earlier tools may not have been directly retrieved through database searches, several pre-2005 instruments were nonetheless identified through systematic reviews and were included in this study.

## Conclusion

Although 13 tools assessed certain visual functions and one tool assessed hearing function, none were specifically developed to evaluate sensory impairment in the context of medication management. Given the prevalence of sensory impairment in older adults and its impact on the safe and effective use of medicines, there is an urgent need for validated tools to assess and support the medication-related needs of this population.

## Supplementary Information

Supplementary Material 1

Supplementary Material 2

## Data Availability

Additional File (1) Search StrategyAdditional File (2) PRISMA Checklist and Critical Appraisal.
