# Supplementary material for: Assessment tools for medication self-management capacity in community-dwelling older adults with sensory impairment: a scoping review
Source: BMC Geriatr. 2025 Nov 25;25:1053. doi: 10.1186/s12877-025-06768-x (PMC12751128; doi:10.1186/s12877-025-06768-x)
Supplement: Supplementary file 1 — Supplementary Material 1 [file 12877_2025_6768_MOESM1_ESM.docx]

**Additional File 2: PRISMA Checklist and Critical Appraisal Tables**

**Preferred Reporting Items for Systematic reviews and Meta-Analyses extension for Scoping Reviews (PRISMA-ScR) Checklist**

| **SECTION** | **ITEM** | **PRISMA-ScR CHECKLIST ITEM** | **REPORTED ON PAGE #** |
| --- | --- | --- | --- |
| **TITLE** | | | |
| Title | 1 | Identify the report as a scoping review. | 1 |
| **ABSTRACT** | | | |
| Structured summary | 2 | Provide a structured summary that includes (as applicable): background, objectives, eligibility criteria, sources of evidence, charting methods, results, and conclusions that relate to the review questions and objectives. | 1 |
| **INTRODUCTION** | | | |
| Rationale | 3 | Describe the rationale for the review in the context of what is already known. Explain why the review questions/objectives lend themselves to a scoping review approach. | 3 |
| Objectives | 4 | Provide an explicit statement of the questions and objectives being addressed with reference to their key elements (e.g., population or participants, concepts, and context) or other relevant key elements used to conceptualise the review questions and/or objectives. | 4 |
| **METHODS** | | | |
| Protocol and registration | 5 | Indicate whether a review protocol exists; state if and where it can be accessed (e.g., a Web address); and if available, provide registration information, including the registration number. | 4 |
| Eligibility criteria | 6 | Specify characteristics of the sources of evidence used as eligibility criteria (e.g., years considered, language, and publication status), and provide a rationale. | 5 |
| Information sources* | 7 | Describe all information sources in the search (e.g., databases with dates of coverage and contact with authors to identify additional sources), as well as the date the most recent search was executed. | 6 |
| Search | 8 | Present the full electronic search strategy for at least 1 database, including any limits used, such that it could be repeated. | 6 |
| Selection of sources of evidence† | 9 | State the process for selecting sources of evidence (i.e., screening and eligibility) included in the scoping review. | 6 |
| Data charting process‡ | 10 | Describe the methods of charting data from the included sources of evidence (e.g., calibrated forms or forms that have been tested by the team before their use, and whether data charting was done independently or in duplicate) and any processes for obtaining and confirming data from investigators. | 6 |
| Data items | 11 | List and define all variables for which data were sought and any assumptions and simplifications made. | 6 |
| Critical appraisal of individual sources of evidence§ | 12 | If done, provide a rationale for conducting a critical appraisal of included sources of evidence; describe the methods used and how this information was used in any data synthesis (if appropriate). | 7 |
| Synthesis of results | 13 | Describe the methods of handling and summarising the data that were charted. | 6 |
| **RESULTS** | | | |
| Selection of sources of evidence | 14 | Give numbers of sources of evidence screened, assessed for eligibility, and included in the review, with reasons for exclusions at each stage, ideally using a flow diagram. | 8 |
| Characteristics of sources of evidence | 15 | For each source of evidence, present characteristics for which data were charted and provide the citations. | 7 |
| Critical appraisal within sources of evidence | 16 | If done, present data on critical appraisal of included sources of evidence (see item 12). | 7 |
| Results of individual sources of evidence | 17 | For each included source of evidence, present the relevant data that were charted that relate to the review questions and objectives. | 8-10 |
| Synthesis of results | 18 | Summarise and/or present the charting results as they relate to the review questions and objectives. | 11-18 |
| **DISCUSSION** | | | |
| Summary of evidence | 19 | Summarise the main results (including an overview of concepts, themes, and types of evidence available), link to the review questions and objectives, and consider the relevance to key groups. | 19-23 |
| Limitations | 20 | Discuss the limitations of the scoping review process. | 23 |
| Conclusions | 21 | Provide a general interpretation of the results with respect to the review questions and objectives, as well as potential implications and/or next steps. | 23 |
| **FUNDING** | | | |
| Funding | 22 | Describe sources of funding for the included sources of evidence, as well as sources of funding for the scoping review. Describe the role of the funders of the scoping review. | 24 |

**MMAT critical appraisal of included studies (n = 10)**

| **Study (First Author, Year, Country)** | **Clear research questions?** | **Data allow addressing research questions?** | **Representative participants?** | **Appropriate measurements?** | **Complete outcome data?** | **Confounders accounted for?** | **Intervention administered as intended?** |
| --- | --- | --- | --- | --- | --- | --- | --- |
| Orwig, 2006 (USA) | Yes | Yes | Yes | Yes | Yes | Yes | Yes |
| Robnett, 2008 (USA) | Yes | Yes | Yes | Yes | Yes | Yes | Yes |
| Lakey, 2009 (USA) | Yes | Yes | Yes | Yes | Yes | Yes | Yes |
| Anderson, 2010 (USA) | Yes | Yes | Yes | Yes | Yes | Yes | Yes |
| Hutchison, 2006 (USA) | Yes | Yes | Yes | Yes | Yes | Yes | Yes |
| Irvine-Meek, 2011 (Canada) | Yes | Yes | Yes | Yes | Yes | Yes | Yes |
| Murphy, 2017 (USA) | Yes | Yes | Yes | Yes | Yes | Yes | Yes |
| Sommerville, 2019 (USA) | Yes | Yes | Yes | Yes | Yes | Yes | Yes |
| Advinha, 2016 (Portugal) | Yes | Yes | Yes | Yes | Yes | Yes | Yes |
| Irvine-Meek, 2010 (Canada) | Yes | Yes | Yes | Yes | Yes | Yes | Yes |

**JBI critical appraisal of included systematic reviews (n = 5)**

| **Study (First Author, Year, Country)** | **Review question clearly stated?** | **Inclusion criteria appropriate?** | **Search strategy appropriate?** | **Sources/resources adequate?** | **Criteria for appraisal appropriate?** | **Critical appraisal by ≥2 reviewers?** | **Methods to minimise extraction errors?** | **Methods to combine studies appropriate?** | **Publication bias assessed?** | **Policy/practice recommendations supported?** | **Directives for new research appropriate?** |
| --- | --- | --- | --- | --- | --- | --- | --- | --- | --- | --- | --- |
| Abed, 2019 (Canada) | Yes | Yes | Yes | Yes | No | No | Yes | Yes | No | No | Yes |
| Elliot, 2010 (Australia) | Yes | Yes | Yes | Yes | No | No | Yes | Yes | No | Yes | Yes |
| Farris, 2008 (USA) | Yes | Yes | Yes | Yes | No | No | Yes | Unclear | No | Yes | Yes |
| Advinha, 2017 (Portugal) | Yes | Yes | Yes | Yes | No | No | Yes | Yes | No | Yes | Yes |
| Elliot, 2009 (Australia) | Yes | Yes | Yes | Yes | No | No | Yes | Yes | No | Yes | Yes |
