# Supplementary material for: Assessment tools for medication self-management capacity in community-dwelling older adults with sensory impairment: a scoping review
Source: BMC Geriatr. 2025 Nov 25;25:1053. doi: 10.1186/s12877-025-06768-x (PMC12751128; doi:10.1186/s12877-025-06768-x)
Supplement: Supplementary file 2 — Supplementary Material 2 [file 12877_2025_6768_MOESM2_ESM.docx]

**Additional File 1: Full Database Search Strategies**

**PubMed/Medline**

Summary of Keywords and Combinations Used for each Search (title, abstract, keyword)

Population – **Older Adults**

Activity – Medication self-management

Outcome - Assessment

Search of PubMed from 2005 to 2023 with fields selected

- #1 "medication therapy management"[MeSH Terms] OR "medication adherence"[MeSH Terms] OR "medication management"[All Fields] OR "medication self management"[All Fields] OR "medicine management"[All Fields] OR "medicine optimisation"[All Fields] OR "medication"[All Fields] (311,280 results)
- #2 “assessment"[Title] OR "assess"[Title] OR "assessing"[Title] OR "psychometrics"[MeSH Terms] OR "validity"[Title] OR "validate"[Title] OR "validating"[Title] OR "psychometric"[Title] OR "psychometrics"[Title] (557,519

results)

- #3 "aged"[MeSH Terms] OR "geriatric"[All Fields] OR "senior"[All Fields] OR "older adult"[All Fields] OR "older people"[All Fields] OR "elderly person"[All Fields] (3,595,440 results)
- #4 (((#1) AND (#2)) AND (#3)) (1987 results)

Summary of Keywords and Combinations Used for each Search (title, abstract, keyword)

Population – **Visually impaired**

Activity – Medication self-management

Outcome - Assessment

Search of PubMed from 2005 to 2023 with fields selected

#1 "medication therapy management"[MeSH Terms] OR "medication adherence"[MeSH Terms] OR "medication management"[All Fields] OR "medication self management"[All Fields] OR "medicine management"[All Fields] OR "medicine optimisation"[All Fields] OR "medication"[All Fields] (311,280 results)

- #2 “assessment"[Title] OR "assess"[Title] OR "assessing"[Title] OR "psychometrics"[MeSH Terms] OR "validity"[Title] OR "validate"[Title] OR "validating"[Title] OR "psychometric"[Title] OR "psychometrics"[Title] (557,519

results)

- #3 "visual impairment"[All Fields] OR "blind"[All Fields] OR "visually handicapped"[All Fields] OR "low vision"[All Fields] OR "sight loss"[All Fields] OR "partial sight"[All Fields] OR "unsighted"[All Fields] OR "purblind"[All Fields] OR ("vision disorders"[MeSH Terms] OR ("vision"[All Fields] AND "disorders"[All Fields]) OR "vision disorders"[All Fields])) (311,634 results)
- #4 (((#1) AND (#2)) AND (#3)) (190 results)

Summary of Keywords and Combinations Used for each Search (title, abstract, keyword)

Population – **Hearing impaired**

Activity – Medication self-management

Outcome - Assessment

Search of PubMed from 2005 to 2023 with fields selected

#1 "medication therapy management"[MeSH Terms] OR "medication adherence"[MeSH Terms] OR "medication management"[All Fields] OR "medication self management"[All Fields] OR "medicine management"[All Fields] OR "medicine optimisation"[All Fields] OR "medication"[All Fields] (311,280 results)

- #2 “assessment"[Title] OR "assess"[Title] OR "assessing"[Title] OR "psychometrics"[MeSH Terms] OR "validity"[Title] OR "validate"[Title] OR "validating"[Title] OR "psychometric"[Title] OR "psychometrics"[Title] (557,519

results)

- #3 "hearing impaired"[All Fields] OR ("hearing loss"[MeSH Terms] OR ("hearing"[All Fields] AND "loss"[All Fields]) OR "hearing loss"[All Fields]) OR "partial hearing"[All Fields] OR "deafness"[All Fields] OR "deaf"[All Fields] OR "deafened"[All Fields] OR "hard of hearing"[All Fields] ("hearing disorders"[MeSH Terms] OR ("hearing"[All Fields] AND "disorders"[All Fields]) OR "hearing disorders"[All Fields])) (140,542 results)
- #4 (((#1) AND (#2)) AND (#3)) (17 results)

**Web of Science – Core Collections**

Summary of Keywords and Combinations Used for each Search (title, abstract, keyword)

Population – **Older Adults**

Activity – Medication self-management

Outcome - Assessment

Search of Web of Science from 2005 to 2023 with fields selected.

- 1 ALL= ("medication therapy management" OR "medication adherence" OR "medication management"[All Fields] OR "medication self management" OR "medicine management" OR "medicine optimisation" OR "medication") (284,952 results)
- 2 TI= (“assessment" OR "assess" OR "assessing" OR "validity" OR "validate" OR "validating" OR "psychometric" OR "psychometrics") (1,030,924 results)

- 3 ALL= ("aged" OR "geriatric" OR "senior" OR "older adult" OR "older people" OR "elderly person") (1,124,457 results)
- 4 #3 AND #2 AND #1 (768 results)

Summary of Keywords and Combinations Used for each Search (title, abstract, keyword)

Population – **Visually impaired**

Activity – Medication self-management

Outcome - Assessment

Search of Web of Science from 2005 to 2023 with fields selected.

- 1 ALL= ("medication therapy management" OR "medication adherence" OR "medication management"[All Fields] OR "medication self management" OR "medicine management" OR "medicine optimisation" OR "medication") (284,952 results)
- 2 TI= (“assessment" OR "assess" OR "assessing" OR "validity" OR "validate" OR "validating" OR "psychometric" OR "psychometrics") (1,030,924 results)

- 3 ALL= ("visual impairment" OR "blind" OR "visually handicapped" OR "low vision" OR "sight loss" OR "partial sight" OR "unsighted" OR "purblind" OR "vision disorders") (483,860 results)
- 4 #3 AND #2 AND #1 (264 results)

Summary of Keywords and Combinations Used for each Search (title, abstract, keyword)

Population – **Hearing impaired**

Activity – Medication self-management

Outcome - Assessment

Search of Web of Science from 2005 to 2023 with fields selected.

- 1 ALL= ("medication therapy management" OR "medication adherence" OR "medication management"[All Fields] OR "medication self management" OR "medicine management" OR "medicine optimisation" OR "medication") (284,952 results)
- 2 TI= (“assessment" OR "assess" OR "assessing" OR "validity" OR "validate" OR "validating" OR "psychometric" OR "psychometrics") (1,030,924 results)

- 3 ALL=("hearing impaired" OR "hearing loss" OR "partial hearing" OR "deafness" OR "deaf" OR "deafened" OR "hard of hearing" OR "hearing disorders") (146,234 results)
- 4 #3 AND #2 AND #1 (15 results)

**EBSCOhost Cinahl**

Summary of Keywords and Combinations Used for each Search (title, abstract, keyword)

Population – **Older Adults**

Activity – Medication self-management

Outcome - Assessment

Search of Cinahl from 2005 to 2023 with fields selected.

- S1 MH "medication therapy management" OR MH "medication adherence" OR "medication management" OR "medication self management" OR "medicine management" OR "medicine optimisation" OR "medication" (144,804 results)
- S2 TI "assessment" OR TI "assess" OR TI "assessing" OR TI "validity" OR TI "validate" OR TI "validating" OR TI "psychometric" OR TI "psychometrics" (183,859 results)

- S3 MH "aged" OR "geriatric" OR "senior" OR "older adult" OR "older people" OR "elderly person" (960,381 results)
- S1 AND S2 AND S3 (809 results)

Summary of Keywords and Combinations Used for each Search (title, abstract, keyword)

Population – **Visually impaired**

Activity – Medication self-management

Outcome - Assessment

Search of Cinahl from 2005 to 2023 with fields selected.

- S1 MH "medication therapy management" OR MH "medication adherence" OR "medication management" OR "medication self management" OR "medicine management" OR "medicine optimisation" OR "medication" (144,804 results)
- S2 TI "assessment" OR TI "assess" OR TI "assessing" OR TI "validity" OR TI "validate" OR TI "validating" OR TI "psychometric" OR TI "psychometrics" (183,859 results)
- S3 "visual impairment" OR "blind" OR "visually handicapped" OR "low vision" OR "sight loss" OR "partial sight" OR "unsighted" OR "purblind" OR MH "vision disorders" (134,632 results)
- S1 AND S2 AND S3 (93 results)

Summary of Keywords and Combinations Used for each Search (title, abstract, keyword)

Population – **Hearing impaired**

Activity – Medication self-management

Outcome - Assessment

Search of Cinahl from 2005 to 2023 with fields selected.

- S1 MH "medication therapy management" OR MH "medication adherence" OR "medication management" OR "medication self management" OR "medicine management" OR "medicine optimisation" OR "medication" (144,804 results)
- S2 TI "assessment" OR TI "assess" OR TI "assessing" OR TI "validity" OR TI "validate" OR TI "validating" OR TI "psychometric" OR TI "psychometrics" (183,859 results)
- S3 "hearing impairment" OR "hearing loss" OR "partial hearing" OR "deafness" OR "deaf" OR "deafened" OR "hard of hearing" OR MH "hearing disorders" (72,696 results)
- S1 AND S2 AND S3 (9 results)

**APA PsycINFO**

Summary of Keywords and Combinations Used for each Search (title, abstract, keyword)

Population – **Older Adults**

Activity – Medication self-management

Outcome - Assessment

Search of APA PsycINFO from 2005 to 2023 with fields selected.

- S1 MA "medication therapy management" OR MA "medication adherence" OR "medication management" OR "medication self management" OR "medicine management" OR "medicine optimization" OR "medication" (192,738 results)
- S2 TI "assessment" OR TI "assess" OR TI "assessing" OR MA "psychometrics" OR TI "validity" OR TI "validate" OR TI "validating" OR TI "psychometric" OR TI "psychometrics" (187,020 results)

- S3 "aged" OR "geriatric" OR "senior" OR "older adult" OR "older people" OR "elderly person" (736,464 results)
- S1 AND S2 AND S3 (637 results)

Summary of Keywords and Combinations Used for each Search (title, abstract, keyword)

Population – **Visually impaired**

Activity – Medication self-management

Outcome - Assessment

Search of APA PsycINFO from 2005 to 2023 with fields selected.

- S1 MA "medication therapy management" OR MA "medication adherence" OR "medication management" OR "medication self management" OR "medicine management" OR "medicine optimization" OR "medication" (192,738 results)
- S2 TI "assessment" OR TI "assess" OR TI "assessing" OR MA "psychometrics" OR TI "validity" OR TI "validate" OR TI "validating" OR TI "psychometric" OR TI "psychometrics" (187,020 results)
- S3 "visual impairment" OR "blind" OR "visually handicapped" OR "low vision" OR "sight loss" OR "partial sight" OR "unsighted" OR "purblind" OR MA "vision disorders" (63,522 results)
- S1 AND S2 AND S3 (266 results)

Summary of Keywords and Combinations Used for each Search (title, abstract, keyword)

Population – **Hearing impaired**

Activity – Medication self-management

Outcome - Assessment

Search of APA PsycINFO from 2005 to 2023 with fields selected.

- S1 MH "medication therapy management" OR MH "medication adherence" OR "medication management" OR "medication self management" OR "medicine management" OR "medicine optimisation" OR "medication" (144,792 results)
- S2 TI "assessment" OR TI "assess" OR TI "assessing" OR MH "psychometrics" OR TI "validity" OR TI "validate" OR TI "validating" OR TI "psychometric" OR TI "psychometrics" (203,354 results)
- S3 "hearing impaired" OR "hearing loss" OR "partial hearing" OR "deafness" OR "deaf" OR "deafened" OR "hard of hearing" OR MA "hearing disorders"  (33,004 results)
- S1 AND S2 AND S3 (2 results)

**Embase**

Summary of Keywords and Combinations Used for each Search (title, abstract, keyword)

Population – **Older Adults**

Activity – Medication self-management

Outcome - Assessment

Search of Embase from 2005 to 2023 with fields selected.

- 1 (“medication Therapy Management" or "medication adherence").sh. or "medication management".af. or "medication self management".af. or "medicine management".af. or "medicine optimization".af. or "medication".af. (512,766 results)
- 2 ("assessment" or "assess" or "assessing" or "validity" or "validate" or "validating" or "psychometric" or "psychometrics").ti. (663,977 results)

- 3 "aged".sh. or "geriatric".af. or "senior".af. or "older adult".af. or "older people".af. or "elderly person".af. (4,025,117 results)
- 1 AND 2 AND 3 (2615 results)

Summary of Keywords and Combinations Used for each Search (title, abstract, keyword)

Population – **Visually impaired**

Activity – Medication self-management

Outcome - Assessment

Search of Embase from 2005 to 2023 with fields selected.

- 1 (“medication Therapy Management" or "medication adherence").sh. or "medication management".af. or "medication self management".af. or "medicine management".af. or "medicine optimization".af. or "medication".af. (512,766 results)
- 2 ("assessment" or "assess" or "assessing" or "validity" or "validate" or "validating" or "psychometric" or "psychometrics").ti. (663,977 results)
- 3 ("visual impairment" or "blind" or "visually handicapped" or "low vision" or "sight loss" or "partial sight" or "unsighted" or "purblind").af. or "vision disorders".sh. (494,268 results)
- 1 AND 2 AND 3 (353 results)

Summary of Keywords and Combinations Used for each Search (title, abstract, keyword)

Population – **Hearing impaired**

Activity – Medication self-management

Outcome - Assessment

Search of Web of Science from 2005 to 2023 with fields selected.

- 1 (“medication Therapy Management" or "medication adherence").sh. or "medication management".af. or "medication self management".af. or "medicine management".af. or "medicine optimization".af. or "medication".af. (512,766 results)
- 2 ("assessment" or "assess" or "assessing" or "validity" or "validate" or "validating" or "psychometric" or "psychometrics").ti. (663,977 results)
- 3 ("hearing impaired" or "hearing loss" or "partial hearing" or "deafness" or "deaf" or "deafened" or "hard of hearing").af. or "hearing disorders".sh. (146,432 results)
- 1 AND 2 AND 3 (15 results)
